# Resveratrol and Its Effects on the Vascular System

**DOI:** 10.3390/ijms20071523

**Published:** 2019-03-27

**Authors:** Johannes M. Breuss, Atanas G. Atanasov, Pavel Uhrin

**Affiliations:** 1 Department of Vascular Biology and Thrombosis Research, Center for Physiology and Pharmacology, Medical University of Vienna, 1090 Vienna, Austria; Johannes.Breuss@meduniwien.ac.at; 2 Department of Molecular Biology, Institute of Genetics and Animal Breeding of the Polish Academy of Sciences, Jastrzębiec, 05-552 Magdalenka, Poland; Atanas.Atanasov@univie.ac.at; 3 Department of Pharmacognosy, Faculty of Life Sciences, University of Vienna, 1090 Vienna, Austria

**Keywords:** resveratrol, clinical studies, cardiovascular disease, vasculoprotective effects

## Abstract

Resveratrol, the phenolic substance isolated initially from *Veratrum grandiflorum* and richly present in grapes, wine, peanuts, soy, and berries, has been attracting attention of scientists and medical doctors for many decades. Herein, we review its effects on the vascular system. Studies utilizing cell cultures and pre-clinical models showed that resveratrol alleviates oxidative stress and inflammation. Furthermore, resveratrol suppresses vascular smooth muscle cell proliferation, promotes autophagy, and has been investigated in the context of vascular senescence. Pre-clinical models unambiguously demonstrated numerous vasculoprotective effects of resveratrol. In clinical trials, resveratrol moderately diminished systolic blood pressure in hypertensive patients, as well as blood glucose in patients with diabetes mellitus. Yet, open questions remain, as exemplified by a recent report which states that the intake of resveratrol might blunt certain positive effects of exercise in older persons, and further research addressing the framework for long-term use of resveratrol as a food supplement, will stay in demand.

## 1. Introduction

Resveratrol, *3,4′,5-trihydroxystilbene*, was first isolated from Veratrum grandiflorum by Takaoka in 1939 [1]. This phenolic substance is present in grapes and wine, as well as in peanuts, soy, berries, and Itadori tea [2,3,4]. Resveratrol is known for its anti-oxidative properties as a scavenger of reactive oxygen species (ROS) such as hydroxyl-, superoxide-, and metal-induced radicals [5,6]. In addition, resveratrol is widely recognized for its anti-aging effects observed in lower organisms [7,8] as well as for its anti-cancer effects [9,10,11,12,13,14,15,16,17]. Lifespan expansion was observed e.g., in *Saccharomyces cerevisiae* [18], *Caenorhabditis elegans* [19], *Drosophila melanogaster* [19] as well as in honey bees [20], and in some short lived vertebrates treated with resveratrol [21]. In mice, resveratrol delayed age-related changes mimicking certain effects of dietary restriction, however, without increasing the life span [22,23]. Many of the anti-aging, as well as anti-cancer effects of resveratrol were assigned to increasing levels of the NAD-dependent deacetylase, designated as “silent mating type information regulation 2 homolog 1”, SIRT1 [24,25].

Resveratrol is in general very well tolerated by human [26,27,28], and only high doses of orally taken resveratrol (2000 mg twice daily) were reported to instigate mild to moderate gastrointestinal symptoms in healthy volunteers [29]. Likewise, no adverse effects were seen for orally administrated resveratrol in experimental animals, at doses 200 mg/kg/day in rats, and 600 mg/kg/day in dogs for 90 days [30]. However, even though the absorption of resveratrol is high, studies in mice, rats, and rabbits demonstrated that resveratrol in the blood degrades relatively quickly, thus reducing its bioavailability [31,32,33]. In rabbits, for instance, its half-life in plasma is only 14 min [34]. Resveratrol’s bioavailability can be partly enhanced by combining it with other phytochemicals e.g., piperine [35], or by using either controlled-release devices or nanotechnological formulations [31,36,37,38]. Resveratrol is, in humans, quickly converted to sulfate- and glucuronide conjugated forms, mainly to *resveratrol-3-O-sulfate*, *resveratrol-4′-O-glucuronide*, and *resveratrol-3-O-glucuronide* [26,39], and these metabolites may provide an intracellular reservoir for the generation of parent resveratrol [40]. Some oncological studies suggested that resveratrol may exhibit biphasic dose responses [41]. Similar concentration-dependent effects were described e.g., in an ischemic heart model, in which resveratrol was cardioprotective when rats were fed for 21 days with low doses (2.5 mg/kg and 25 mg/kg), while it was not protective when fed in high (100 mg/kg) doses [42].

The notion that resveratrol may be beneficial for the human vasculature arose from the epidemiological data and so-called “French paradox”. According to those findings, the French population, in spite of a high intake of saturated fat, is at rather low risk of cardiovascular diseases, and the protective effects were assigned to relatively high wine consumption [43]. In later studies, resveratrol was designated as a substance partly accountable for such protective effects [44,45,46,47,48]. Studies showed that the vasculoprotective benefits of resveratrol are mediated by different mechanisms, including the lowering of oxidative stress and inflammation, enhancing metabolic capacity, increasing NO synthesis by endothelial cells, suppressing vascular smooth muscle cell (VSMC) proliferation and promoting autophagy. Furthermore, resveratrol was investigated for its ability to prevent cellular senescence, but accomplished works showed conflicting results. Below we discuss studies performed in cell-cultures, pre-clinical models, and human clinical trials, investigating in detail different aspects of resveratrol effects on the vasculature.

## 2. In Vitro Studies and Studies in Pre-clinical Models on Resveratrol in the Context of Cardiovascular Diseases

### 2.1. Resveratrol Reduces Oxidative Stress, Alleviates Inflammation, and Increases NO Synthesis

Early studies showed that resveratrol suppresses oxidation of human low-density lipoprotein, (LDL) [49], as well as reduces lipid peroxidation [50]. Feeding with resveratrol decreased lethality in mice challenged by lipopolysaccharide (LPS) treatment [51]. Resveratrol food supplementation increased activities of the anti-oxidative enzymes superoxide dismutase and glutathione peroxidase in rat myocardium and aorta [52]. Such treatment also enhanced the expression of the protective nuclear factor erythroid 2-related factor 2 (Nrf2) and reduced the mortality of mice exposed to catecholamine administration [52]. Resveratrol furthermore reduces the oxidative load via suppressing NADPH oxidase-mediated production of ROS, and via enhancing the expression of various antioxidant enzymes [53]. In *Caenorhabditis elegans*, resveratrol diminished oxidative stress induced by radiation, in two different studies [54,55].

Anti-inflammatory effects of resveratrol include the inhibition of the pro-inflammatory enzyme cyclooxygenase-1 (COX-1), leading to the suppression of synthesis of proinflammatory eicosanoids [56,57]. The anti-inflammatory effects can be mediated by SIRT1 that, via deacetylation, suppresses the major inflammatory transcription factor nuclear factor-κB, NF-κB [58]. In mouse skin, resveratrol moderates the phorbol ester-induced pro-inflammatory NF-κB and AP-1 pathways, and suppresses expression of COX-2 [59,60]. Resveratrol also alleviates the induction of inflammation in mice fed with a high-fat diet [61].

Further vasculoprotective benefits of resveratrol enclose an increase in the formation of the vasculoprotective nitric oxide (NO). Such effect was observed in human umbilical vein endothelial cells (HUVECs) and HUVEC-derived EA.hy 926 cells, where resveratrol, similarly as an alcohol-free red wine polyphenol extract, induced the expression of endothelial nitric oxide synthase (eNOS) thus leading to an increased NO synthesis [62,63]. These effects of resveratrol in endothelial cells are fostered by the activation of estrogen receptor (ER)- and mitogen-activated protein kinase (MAPK) signaling [64], and additionally mediated by SIRT1, and the transcription factors FOXO1 and FOXO3a [65]. The enhancement of eNOS synthesis and NO production that was facilitated in HUVECs by ER and peroxisome proliferator-activated receptor α (PPARα), became robust during repeated and long-term treatment with resveratrol [66]. Resveratrol also induced the expression of the vasculoprotective transcription factor Krüppel-like factor 2 (KLF2) via SIRT1 activation [67]. The protective effects of resveratrol mediated by increased NO synthesis were not only seen in cell culture, but also demonstrated in animal models. For example, the feeding of hypercholesterolemic rabbits with resveratrol, or the administration of red wine or dealcoholized red wine, improved the functionality of the endothelium, as detected by flow-mediated dilation measurements in the femoral artery [44]. These alterations were accompanied by diminished endothelin 1 (ET-1)- and enhanced NO levels in plasma [44]. The intake of resveratrol stimulated the expression of eNOS, of inducible NO synthase (iNOS) and of vascular endothelial growth factor (VEGF) in the heart of experimental rats [68].

### 2.2. Resveratrol Enhances Aerobic Capacity of Muscles and Alleviates Endothelial Dysfunction Induced by Diabetes and Obesity

The oral administration of resveratrol to mice fed with a high-fat diet enhanced their aerobic capacity, as demonstrated by prolonged running times and increased muscle oxygen consumption [69]. Such changes were associated with an increased expression of genes involved in oxidative phosphorylation and mitochondrial biogenesis (in the heart, muscles, and brown adipose tissue), and were linked to SIRT1 expression controlling energy and metabolic homeostasis [69]. Resveratrol also maintained the mitochondrial function and stimulated the mitochondrial biogenesis in regulatory T-cells in mice fed with a high-fat diet, pointing to the fact that the effects of resveratrol on oxidative metabolisms of mitochondria are not restricted to certain tissues [70].

In addition, resveratrol counteracted some harmful effects of diet-induced obesity and reduced insulin resistance in animal models. Its administration to middle-aged mice fed with a high-calorie diet improved the motor function and enlarged the number of mitochondria [71]. Such treatment also affected numerous signaling pathways and resulted in enhanced insulin sensitivity, decreased levels of insulin-like growth factor-1, and raises in peroxisome proliferator-activated receptor-γ coactivator 1α (PGC-1α) and AMP-activated protein kinase (AMPK) activity [71]. The latter protein is known as an important nutrient and energy sensor maintaining energy homeostasis of the organism [72].

In the myocardium of rats with streptozotocin-induced diabetes, resveratrol stimulated GLUT-4 translocation to the cell membrane, and this alteration was mediated by the AMPK/Akt/eNOS pathway [73]. Resveratrol also delayed vascular aging in rats without prolonging their life span [74]. In rhesus monkeys fed with a high-fat/high-sugar diet, a two-year supplementation with resveratrol reduced the adipocyte size as well as the inflammatory response in the adipose tissues [75]. Such treatment also improved insulin signaling [75]. The protective effects of resveratrol-administration in rhesus monkeys fed with a high-fat/high-sugar diet also included the prevention of beta-cell dedifferentiation [76]. Resveratrol furthermore suppressed, in rhesus monkeys fed with high-fat/high-sucrose diet, inflammation and the stiffening of the arterial wall [77]. The feeding of rats with resveratrol also increased SIRT1 and adiponectin levels in their serum [78].

In cultured human coronary arterial endothelial cells, resveratrol-treatment alleviated mitochondrial oxidative stress induced by high-glucose [79]. These protective effects were boosted by overexpressing SIRT1, and diminished by its downregulation [79]. In human coronary arterial endothelial cells, resveratrol furthermore enhanced mitochondrial mass and mitochondrial DNA [80,81]. Such effects were dependent on the expression of SIRT1 [80] and of the antioxidant transcription factor Nrf2 [81]. The impact of these findings was further reinforced by the fact that prolonged resveratrol-treatment normalized mitochondrial biogenesis in the aortas of type 2 diabetic- (db/db) [80], as well as of wild-type mice fed with a high-fat diet [81]. Various studies also showed that resveratrol inhibits lipogenesis as well as differentiation of pre-adipocytes to mature adipocytes [82,83,84]. Resveratrol furthermore lessened consequences of experimentally-induced diabetic neuropathy in rats [85]. Specifically, a two-week treatment of rats with resveratrol that started six week after diabetes induction by streptozotocin, significantly counteracted the reduction in motor nerve conduction velocity and in nerve blood flow, and diminished thermal hyperalgesia of rats [85]. Protective effects of resveratrol intake were also reported in db/db mice and included decrease in blood glucose, free fatty acid and triglycerides levels [86]. These effects were associated with the increased expression of GLUT4 protein in the skeletal muscle, and the activation of AMPK and its downstream targets [86].

### 2.3. Resveratrol Promotes *Autophagy* and Might Protect Against Cellular *Senescence*

Resveratrol stimulated autophagy, the process of removing and recycling damaged cellular components, such as organelles, membranes or proteins, in different cell-types [87], e.g., in endothelial cells exposed to tumor necrosis factor-α (TNF-α), where its effects were mediated via the cAMP signaling pathway [88]. As it is known that insufficiently operating autophagy promotes mitochondrial dysfunction and oxidative stress [89,90,91], the above studies provide further support for the vasculoprotective role of resveratrol.

Conflicting results were, however, reported on the role of resveratrol in mediating cellular senescence, the state in which cells have lost their capacity to divide but secrete inflammatory cytokines [92,93,94,95]. Such proinflammatory senescent cells, accumulating with age in the affected tissues, contribute to the loss of tissue homeostasis, and to the development of age-associated pathologies [93]. In an attempt to investigate the presumed beneficial effects of resveratrol, surprisingly, the continuous treatment of HUVECs with 10 µM resveratrol during extended cell-propagation, resulted in the premature replicative senescence of these cells. They were found arrested in the S phase of the cell cycle, caused, apparently, by elevated levels of ROS [96]. Such increased ROS production, detected in HUVECs treated with resveratrol, was mediated via the NADPH oxidases Nox1 and Nox4, and the blocking of these enzymes prevented the resveratrol-induced cellular senescence [96]. On the other hand, another group reported that resveratrol protects human endothelium against H_2_O_2_ induced senescence, specifically by reducing H_2_O_2_-induced oxidative stress via activating SIRT1 [97]. In line with the view of an overall protective effect of resveratrol are data obtained in vivo, in rats fed with a high-fat/high-sucrose diet and concomitantly treated with resveratrol [98]. In the aortas of these animals, resveratrol prevented the rise in senescent cells [98]. In addition, resveratrol lessened the induction of senescence in bovine aortic endothelial cells cultured in high-glucose medium [98]. The protective effects of resveratrol-treatment in this study were assigned to its ability to diminish an increase in NADPH oxidase subunit p47phox expression and counteract a decrease in SIRT1 expression caused by high-fat/high-sucrose treatment [98].

### 2.4. Resveratrol Alleviates Oxidative Stress in Cardiac Cells and Macrophages

Resveratrol also affected other cell-types. In the H9C2 cell line derived from embryonic rat heart ventricle, it stimulated the expression of cellular antioxidants, as well as phase 2 enzymes, and diminished intracellular ROS levels that had been induced by oxidative or electrophilic injury [99]. In LPS-stimulated macrophages resveratrol suppressed their transition to foam cells and alleviated oxidative stress, by suppressing Nox1 expression and ROS production [100]. Further changes mediated by resveratrol in LPS-stimulated macrophages, included a diminished expression of the chemoattractant monocyte chemotactic protein-1, MCP-1 [100].

### 2.5. Resveratrol Suppresses VSMC Proliferation and Platelet Aggregation

Dysregulated and excessive VSMC proliferation is known to contribute to the development of atherosclerosis, as well as restenosis after vascular surgery [101,102]. Several in vitro and in vivo studies demonstrated that resveratrol diminishes VSMC proliferation. The treatment of VSMCs, derived from spontaneously hypertensive rats, with resveratrol suppressed advanced glycation end-products (AGEs)-induced proliferation of VSMCs, as well as reduced collagen synthesis [103]. Resveratrol also inhibited the serum-induced proliferation of rat aortic-SMCs, and this effect was synergistically enhanced in the presence of other polyphenols present in red wine (quercetin, (+)-catechin and ethyl gallate) [104]. Resveratrol furthermore suppressed oxidized low-density lipoprotein (ox-LDL)-induced proliferation of cultured bovine aortic SMCs [105]. Mechanistically, the inhibitory effects of resveratrol were associated with the suppression of MAPK ERK1/2 and the weakening oxLDL-induced ROS and H_2_O_2_ production [105]. In addition, resveratrol attenuated the proliferation of human coronary SMCs induced by ET-1, and these effects were associated with the activation of kinase-G and suppressing ERK1/2 activation [106]. The subcutaneous administration of resveratrol to neonatal rats placed into a hypobaric hypoxic chamber alleviated remodeling of the right ventricle and of the pulmonary artery [107], a hallmark of pulmonary hypertension [108]. In vitro, resveratrol diminished the hypoxia-induced proliferation of human pulmonary artery-SMCs via inhibiting arginase II, an enzyme known to be upregulated in patients with pulmonary hypertension, and these effects were mediated via affecting PI3K-Akt signaling [107]. Resveratrol furthermore attenuated the homocysteine-induced proliferation of VSMCs, by reducing hypermethylation of phosphatase and tensin homologue on chromosome 10, PTEN [109]. In addition, in rats subjected to a chronic myocardial ischemia model, resveratrol induced the expression of Krüppel-like factor 15 (KLF15), known to play a protective role in the ischemic myocardium [110].

Importantly, some vasculoprotective effects of resveratrol could be attributed to inhibiting platelet aggregation, as exacerbated platelet aggregation/activation is an important risk factor for atherosclerosis [111]. The treatment of human platelets with resveratrol significantly suppressed their aggregation induced by collagen, thrombin, or ADP [112,113]. The administration of resveratrol (4 mg/kg/day) to rabbits fed with a high-cholesterol diet diminished ADP-induced platelet aggregation ex vivo [112].

### 2.6. Resveratrol has Protective Effects in Heart-, Lung-, Brain-, and Skin Injury Models

Resveratrol also was protective in heart injury/infarction- and hemorrhagic lung-, brain-, and skin injury pre-clinical models, and these effects were mediated via suppressing oxidative stress and inflammation, increasing NO levels, mediating ion channel activation, and promoting autophagy.

The beneficial effects of resveratrol on cardiomyocytes seen in an ischemia-reperfusion model included the suppression of superoxide levels, the activation of potassium channels, as well as an increase in endothelium-dependent vasodilatation [114]. In rats fed with a hypercholesterolemic diet, and subjected to experimentally induced myocardial infarction, the oral intake of resveratrol amended certain cardiologic parameters such as ejection fraction and fractional shortening and fostered neovascularization in the injured myocardium [115]. These benefits were associated with increased expressions of hemeoxygenase-1 (HO-1), eNOS and VEGF in resveratrol-treated animals [115]. Furthermore, a three week feeding of rats with resveratrol was protective against ischemia/reperfusion injury, and normalized an altered microRNA pattern [116]. Resveratrol enhanced survival, hemodynamics and energetics in rats, in a model of hypertension leading to heart failure [117]. Specifically, Dahl salt-sensitive rats had been fed with a high-salt diet, and resveratrol was administrated for eight weeks after inducing hypertension and cardiac hypertrophy. Such treatment increased the rats’ survival, by counteracting cardiac dysfunction [117]. Observed changes also included the preservation of mitochondrial mass, and an increase in PPARα-expression [117].

The intraperitoneal administration of resveratrol to newborn rats subjected to hyperoxia-induced lung injury significantly diminished TNF-α levels and increased the expression of crucial antioxidants glutathione- and superoxide dismutase (SOD) [118]. Resveratrol also suppressed inflammation and fibrosis in the lungs of neonatal rats exposed to hyperoxia-induced oxidative stress, and these effects were accompanied by inhibition of Wnt/beta-catenin signaling [119]. The intraperitoneal administration of resveratrol prevented the expression of inflammatory markers in an acute lung injury model in rats [120].

The prolonged administration of resveratrol in rats was also neuroprotective, as such treatment partially prevented tissue damage in the olfactory cortex and the hippocampus, caused by systemic injection of the excitotoxin kainic acid [121]. Resveratrol was also found neuroprotective in a rat stroke model where its benefits were assigned to suppressing of phosphodiesterases and influencing the cAMP/AMPK/SIRT1 pathway [122]. Treatment of rats with resveratrol immediately after subarachnoid hemorrhagic injury, reduced mortality and brain edema [123]. In this model, the protective effects of resveratrol were mediated by the Akt/mTOR pathway [123].

Finally, the prolonged local treatment of skin wounds of rats with resveratrol caused accelerated wound healing due to enhanced vascularization [124]. These effects were mediated via stimulation of the AMPK pathway [124].

## 3. Clinical Studies on Resveratrol in the Context of Cardiovascular Diseases

Many clinical studies investigated the effects of resveratrol intake in the context of cardiovascular diseases [125]. These studies differ widely in the used amounts of resveratrol (ca. 5 to 5,000 mg/day), and in treatment periods (ranging from s couple of days to months). Results of some of these works are highlighted in Table 1.

The controlled drinking of wine (300 mL/day) for 15 days by test persons led to an increase of plasma resveratrol concentration [126]. In addition, the treatment of platelets with resveratrol in vitro enhanced the activity of platelet NO synthase (NOS) and caused a decrease in phosphorylation of the proinflammatory p38 MAPK, and reduction in the NADPH oxidase activity [126].

The acute treatment of healthy volunteers with resveratrol enhanced cerebral blood flow [127]. Prolonged treatment of Alzheimer disease patients with orally administrated resveratrol (500 mg once daily with dose escalation until a final dose of 1000 mg twice daily) caused a decrease in matrix metalloproteinase 9 (MMP-9) levels, as well as improved responsiveness of microglia/macrophages in the cerebrospinal fluid [128]. In addition, in these subjects, reduced plasma levels of the proinflammatory factors, interleukin (IL)-1R4, IL-12p40, IL-12p70, and TNF-α were found [128].

Clinical trials investigated the effects of resveratrol in obese patients with metabolic syndrome and focused mainly on the investigation of metabolic changes and parameters, such as body mass, body mass index (BMI), blood pressure, lipid profile, glucose, and inflammation status. For example, a 30 day resveratrol intake, by obese humans, prompted metabolic changes in muscles such as the activation of AMPK, and increase in SIRT1- and PGC-1α protein levels [129]. In addition, plasma glucose, triglycerides, and inflammation markers, became reduced after resveratrol-treatment, and, the metabolic changes observed in this study mimicked the effects of calorie restriction [129]. Pooled analysis of 21 clinical studies that recruited overweight and obese study participants, showed that resveratrol-treatment significantly decreased total cholesterol, systolic blood pressure, and fasting glucose [130]. These effects were more prominent in individuals ingesting more than 300 mg of resveratrol per day [130]. A more recent study evaluating 28 randomized clinical trials accomplished until April 2018 revealed the significant impact of resveratrol intake on a decrease in body weight, BMI, and waist circumference [131]. These effects were more prominent in studies that lasted longer than three months and were performed on obese people [131]. Similar conclusions were drawn in a meta-analysis that encompassed 36 randomized clinical trials accomplished until July 2018. Here, resveratrol intake significantly decreased body weight, BMI, fat mass, and waist circumference [132], demonstrating altogether the positive effects of resveratrol supplementation on weight loss. Although data of how resveratrol affects blood pressure are rather heterogeneous and partly controversial, positive blood pressure-lowering effects were concluded in a recent analysis of 17 randomized controlled clinical trials, in either individuals receiving more than 300 mg per day of resveratrol, or in diabetic patients [133]. Meta-analyses of randomized clinical trials furthermore concluded that the intake of resveratrol does not influence total cholesterol-, low-density lipoprotein (LDL)-, and high density lipoprotein (HDL) cholesterol levels, but it may decrease triglycerides [134]. However, no beneficial effects of resveratrol intake on lowering the inflammatory marker C-reactive protein (CRP) were found, neither in a meta-analysis of randomized controlled trial [135] nor in a later accomplished randomized placebo-controlled clinical trial [136]. These data are, however, in contrast with two more recent meta-analyses concluding the CRP-lowering effects of resveratrol use [137,138].

Treatment with resveratrol alleviated some clinical parameters of diabetes mellitus in several studies. For example, the daily consumption of a resveratrol-enriched grape extract for one year reduced the expression of pro-inflammatory cytokines CCL3, IL-1β, and TNF-α, and modified the pattern of inflammatory-related microRNAs in peripheral blood mononuclear cells of type 2 diabetes patients and hypertensive patients with coronary artery disease [139]. In another study, a 30 day resveratrol administration to obese human subjects, suppressed postprandial glucagon levels [140]. A three month intake of resveratrol (250 mg per day) by type 2 diabetic patients, reduced systolic blood pressure, as well as total cholesterol levels, even though it did not change body weight and LDL and HDL cholesterol levels in comparison with a placebo group [141]. Treatment of type 2 diabetic patients with resveratrol (one gram per day for 45 days) reduced fasting blood glucose levels as well as HbA1c and improved insulin resistance [142]. Meta-analyses of randomized placebo controlled clinical trials found positive effects of prolonged resveratrol intake in type 2 diabetes mellitus-patients on lowering systolic blood pressure [143], triglyceride levels [144] as well as HbA1c and creatinine levels [143]. No effects of resveratrol intake on fasting glucose, diastolic blood pressure, insulin, LDL, and HDL cholesterol levels were found here [143], contrary to other meta-analyses that reported glucose lowering effects of resveratrol administration [145]. The meta-analysis of six studies involving patients treated with resveratrol did not find blood pressure lowering effects of resveratrol [146]. Yet, reduction in systolic blood pressure was seen in a subgroup treated with a high dose of resveratrol (≥ 150 mg per day) [146].

Contrary to exercise training, resveratrol intake surprisingly did not improve the metabolic or inflammatory status in skeletal muscles of elderly persons, and the authors furthermore concluded that the use of resveratrol may not be beneficial for the cardiovascular system in aged man [147,148,150]. These studies stimulated intense discussions in the scientific and medical community [151,152,153,154,155,156,157]. The effects of resveratrol intake and exercise are currently being investigated in a clinical trial involving 60 sedentary persons aged >65 years [149].

Beyond the conditions described above, future clinical studies might also focus on investigating the effects of resveratrol in ischemic heart disease and/or atrial fibrillation [158,159,160,161]. In addition, future studies might also be needed to determine in more detail the effects of resveratrol on chronic obstructive pulmonary disease [162], representing a serious clinical problem [108].

Additional reviews highlighting different aspects of resveratrol on the vasculature are e.g., References [163,164,165,166,167,168,169,170,171,172,173,174].

## 4. Conclusions

Numerous in vitro studies and studies in preclinical models demonstrated vasculoprotective effects of resveratrol. Resveratrol is well tolerated, both in experimental animals and in humans, and positive effects of resveratrol observed in pre-clinical models included e.g., alleviation of oxidative stress and inflammation, enhancement of metabolic capacity, increased NO synthesis, suppression of VSMC proliferation, and elevation of autophagy. Human clinical studies markedly differ in the doses of administrated resveratrol as well as in duration of the treatment. Overall, the most pronounced effects of resveratrol included reduction in body weight in obese patients and partly diminishing systolic blood pressure as well as fasting blood glucose and HbA1c levels in patients with diabetes mellitus in some clinical trials. Currently intensively studied topics include e.g., the evaluation of the effects of a combined use of resveratrol and exercise in older sedentary persons, as well as the optimization of the dose- and time frame of resveratrol use.

## Figures and Tables

**Table 1:** Outcome of clinical studies involving intake of resveratrol.

Type of the Study/Number of Probands	Clinical Outcome	References
Healthy volunteers (N = 20) tested before and after 15 days of controlled wine consumption (300 mL/day)	Increase in resveratrol concentration in plasma after wine consumption. Enhancement of platelet NO synthase (NOS) activity, decrease in phosphorylation of p38 MAPK and reduction in NADPH oxidase activity after treatment of platelets with resveratrol *in vitro*	[126]
Healthy volunteers (N = 22) who received orally placebo and two doses of resveratrol (250 and 500 mg) on separate days	Enhancement in cerebral blood-flow after resveratrol intake (cognitive functions stayed not affected)	[127]
Patients with mild-to-moderate Alzheimer’s disease (N = 119) randomized to placebo or resveratrol group and treated for 52 weeks (the latter group was receiving orally 500 mg of resveratrol once daily with dose escalation until a final dose of 1000 mg twice daily)	Presence of low nanomolar concentration of resveratrol in the cerebrospinal fluid of resveratrol- treated group; a 50% decrease of MMP-9 level and increase in activation of microglia/macrophages; reduced plasma levels of proinflammatory interleukin (IL)-1R4, IL-12p40, IL-12p70, and TNF; weight loss in resveratrol-treated group	[128]
Healthy obese men (N = 11) treated with placebo and subsequently with 150 mg/day resveratrol for 30 days	Significant reduction in sleeping- and resting metabolic rates due to resveratrol-treatment. Activation of AMPK, enhancement of SIRT1 and PGC-1α protein levels in muscle, and decrease in plasma glucose, triglycerides levels and inflammation markers	[129]
Pooled analysis of 21 clinical studies that included overweight and obese human	Resveratrol-treatment significantly decreased total cholesterol, systolic blood pressure and fasting glucose, effects more pronounced in individuals ingesting more than 300 mg of resveratrol per day	[130]
Meta-analysis of 28 randomized controlled trials	Significant reduction in body weight, BMI and waist circumference; effects of resveratrol most prominent in obese patients and in trials longer than three months	[131]
Meta-analysis of 36 randomized controlled trials	Significant reduction in body weight, BMI, fat mass and waist circumference; no significant effect of resveratrol intake on leptin and adiponectin levels	[132]
Meta-analysis of 17 randomized controlled trials	Intake of resveratrol did not significantly affect systolic, diastolic or mean blood pressure. However, significant blood pressure lowering effects were found in individuals treated with resveratrol daily at dosage ≥ 300 mg per day and in diabetic patients	[133]
Meta-analysis of 21 randomized clinical trials	Resveratrol could not significantly change total cholesterol, LDL and HDL cholesterol levels, but it might decrease blood triglycerides levels	[134]
Meta-analysis of 10 randomized clinical trials	No changes in C-reactive protein (CRP) blood levels. In addition, no alterations in total cholesterol, LDL cholesterol and triglycerides plasma levels upon resveratrol treatment	[135]
A randomized placebo-controlled clinical trial including middle-aged men (N = 74) with metabolic syndrome receiving daily 1000 mg, 150 mg of resveratrol, or placebo for 16 weeks	No lowering of CRP, interleukin 6, or soluble urokinase plasminogen activator receptor plasma levels. No change in analyzed inflammatory gene expression in adipose and muscle tissues. No effect on blood pressure. A striking increase in total cholesterol and LDL cholesterol plasma levels in resveratrol-treated compared to placebo-treated men	[136]
Meta-analysis of 15 randomized clinical trials (N = 658)	Resveratrol decreased serum CRP levels; no significant change in serum IL-6 and TNF-α levels	[137]
Meta-analysis of 17 randomized clinical trials (N = 736)	Significant reduction in CRP- and TNF-α levels; no significant change in IL-6 in serum upon resveratrol treatment	[138]
Type 2 diabetes mellitus and hypertensive patients consuming daily resveratrol’s enriched (8 mg) grape extract	Reduced expression of pro-inflammatory cytokines CCL3, IL-1β and TNF-α; modified pattern of inflammatory-related microRNAs in peripheral blood mononuclear cells due to resveratrol enriched grape extract consumption	[139]
Obese human subjects (N = 10) treated with resveratrol for 30 days	Decrease in postprandial glucagon levels due to resveratrol’s intake, no changes in fasting plasma glucagon levels	[140]
A randomized clinical trial including a three month treatment of type 2 diabetic patients (N = 62) with 250 mg per day of resveratrol or placebo	Reduction in systolic blood pressure and total cholesterol levels, no change in body weight and LDL and HDL cholesterol levels in comparison with placebo group	[141]
Type 2 diabetic patients taking resveratrol orally (1 gram per day for 45 days) in the presence of standard antidiabetic treatment (N = 34 resveratrol, N = 32 placebo)	Reduction in systolic blood pressure, fasting blood glucose and HbA1c levels and improvement in insulin resistance in the resveratrol group	[142]
Meta-analyses of six randomized controlled clinical trials of type 2 diabetes mellitus patients (N=104 resveratrol, N = 92 placebo)	Reduction in systolic blood pressure, HbA1c, and creatinine levels in the resveratrol group. No change in other clinical parameters (fasting glucose, insulin resistance, triglycerides, LDL and HDL cholesterol)	[143]
Meta-analysis of 10 randomized clinical trials including patients with type 2 diabetes mellitus (N = 363)	Prolonged treatment of diabetic patients (≥ 6 months) with resveratrol reduced triglyceride levels	[144]
Meta-analysis of nine randomized clinical trials including patients with type 2 diabetes mellitus (N = 283)	Significant improvement of the fasting plasma glucose and insulin levels (especially at a dose of resveratrol ≥ 100 mg per day) as well as reduction of blood pressure. No significant changes in HbA1c, LDL and HDL cholesterol	[145]
Meta-analysis of six studies involving patients treated with resveratrol (N = 247)	No significant reduction in systolic blood pressure in the whole resveratrol group. Reduction in systolic blood pressure in a subgroup treated at a high dose of resveratrol (≥ 150 mg per day). No changes in diastolic blood pressure	[146]
Healthy physically inactive 65 years old men subjected to eight week exercise training and additionally taking 250 mg per day of trans-resveratrol (N = 14) or placebo (N = 13)	In the trained placebo group: a ~45% higher increase in maximal oxygen uptake when compared to the trained resveratrol group. Reduction in the mean arterial pressure detected only in the trained placebo group. Lower interstitial levels of vasodilator prostacyclin and higher levels of muscle thromboxane synthase in the trained resveratrol group than in trained placebo group. Reduction in LDL, total cholesterol/HDL ratio and triglyceride concentrations in the blood detected only in the trained placebo group (not in the trained resveratrol group)	[147]
Healthy physically inactive 65 years old men subjected to eight week intense exercise training and additionally taking 250 mg per day of trans-resveratrol (N = 14) or placebo (N = 13). Non trained group received 250 mg per day of trans-resveratrol (N = 9) or placebo (N = 7)	In the trained placebo group: a ~20% increase in the ratio of capillary to muscle fibers as well as increase in levels of VEGF, VEGF receptor-2, and tissue inhibitor of matrix metalloproteinase (TIMP-1). In the trained resveratrol group: no increase in the ratio of capillary to muscle fibers as well as no increase in VEGF levels	[148]
60 sedentary persons aged > 65 years will be exercising three times weekly during three months. Participants are being/will be assigned to three groups: consuming 1) placebo, 2) 250 mg/day resveratrol, or 3) 1000 mg/day resveratrol.	This running clinical trial should provide an important information on the skeletal muscle mitochondrial function induced by a combined use of resveratrol and exercise in older sedentary persons	[149]

## References

[1] Takaoka M. (1939). Resveratrol, a new phenolic compound, from Veratrum grandiflorum. J. Chem. Soc. Jpn..

[2] Burns J., Yokota T., Ashihara H., Lean M.E., Crozier A. (2002). Plant foods and herbal sources of resveratrol. J. Agric. Food Chem..

[3] Stervbo U., Vang O., Bonnesen C. (2007). A review of the content of the putative chemopreventive phytoalexin resveratrol in red wine. Food Chem..

[4] Sales J.M., Resurreccion A.V. (2014). Resveratrol in peanuts. Crit. Rev. Food Sci. Nutr..

[5] Leonard S.S., Xia C., Jiang B.H., Stinefelt B., Klandorf H., Harris G.K., Shi X. (2003). Resveratrol scavenges reactive oxygen species and effects radical-induced cellular responses. Biochem. Biophys. Res. Commun..

[6] Truong V.L., Jun M., Jeong W.S. (2018). Role of resveratrol in regulation of cellular defense systems against oxidative stress. BioFactors.

[7] Bhullar K.S., Hubbard B.P. (2015). Lifespan and healthspan extension by resveratrol. Biochim. Biophys. Acta.

[8] Li Y.R., Li S., Lin C.C. (2018). Effect of resveratrol and pterostilbene on aging and longevity. BioFactors.

[9] Carter L.G., D’Orazio J.A., Pearson K.J. (2014). Resveratrol and cancer: focus on in vivo evidence. Endocr.-Relat. Cancer.

[10] Shrotriya S., Agarwal R., Sclafani R.A. (2015). A perspective on chemoprevention by resveratrol in head and neck squamous cell carcinoma. Adv. Exp. Med. Biol..

[11] Xu Q., Zong L., Chen X., Jiang Z., Nan L., Li J., Duan W., Lei J., Zhang L., Ma J. (2015). Resveratrol in the treatment of pancreatic cancer. Ann. N. Y. Acad. Sci..

[12] Varoni E.M., Lo Faro A.F., Sharifi-Rad J., Iriti M. (2016). Anticancer Molecular Mechanisms of Resveratrol. Front. Nutr..

[13] Kim C.W., Hwang K.A., Choi K.C. (2016). Anti-metastatic potential of resveratrol and its metabolites by the inhibition of epithelial-mesenchymal transition, migration, and invasion of malignant cancer cells. Phytomedicine.

[14] Jiang Z., Chen K., Cheng L., Yan B., Qian W., Cao J., Li J., Wu E., Ma Q., Yang W. (2017). Resveratrol and cancer treatment: updates. Ann. N. Y. Acad. Sci..

[15] Alamolhodaei N.S., Tsatsakis A.M., Ramezani M., Hayes A.W., Karimi G. (2017). Resveratrol as MDR reversion molecule in breast cancer: An overview. Food Chem. Toxicol..

[16] Ko J.H., Sethi G., Um J.Y., Shanmugam M.K., Arfuso F., Kumar A.P., Bishayee A., Ahn K.S. (2017). The Role of Resveratrol in Cancer Therapy. Int. J. Mol. Sci..

[17] Espinoza J.L., Kurokawa Y., Takami A. (2018). Rationale for assessing the therapeutic potential of resveratrol in hematological malignancies. Blood Rev..

[18] Howitz K.T., Bitterman K.J., Cohen H.Y., Lamming D.W., Lavu S., Wood J.G., Zipkin R.E., Chung P., Kisielewski A., Zhang L.L. (2003). Small molecule activators of sirtuins extend Saccharomyces cerevisiae lifespan. Nature.

[19] Wood J.G., Rogina B., Lavu S., Howitz K., Helfand S.L., Tatar M., Sinclair D. (2004). Sirtuin activators mimic caloric restriction and delay ageing in metazoans. Nature.

[20] Rascon B., Hubbard B.P., Sinclair D.A., Amdam G.V. (2012). The lifespan extension effects of resveratrol are conserved in the honey bee and may be driven by a mechanism related to caloric restriction. Aging.

[21] Valenzano D.R., Terzibasi E., Genade T., Cattaneo A., Domenici L., Cellerino A. (2006). Resveratrol prolongs lifespan and retards the onset of age-related markers in a short-lived vertebrate. Curr. Biol..

[22] Pearson K.J., Baur J.A., Lewis K.N., Peshkin L., Price N.L., Labinskyy N., Swindell W.R., Kamara D., Minor R.K., Perez E. (2008). Resveratrol delays age-related deterioration and mimics transcriptional aspects of dietary restriction without extending life span. Cell Metab..

[23] Barger J.L., Kayo T., Vann J.M., Arias E.B., Wang J., Hacker T.A., Wang Y., Raederstorff D., Morrow J.D., Leeuwenburgh C. (2008). A low dose of dietary resveratrol partially mimics caloric restriction and retards aging parameters in mice. PLoS ONE.

[24] Herranz D., Munoz-Martin M., Canamero M., Mulero F., Martinez-Pastor B., Fernandez-Capetillo O., Serrano M. (2010). Sirt1 improves healthy ageing and protects from metabolic syndrome-associated cancer. Nat. Commun..

[25] Hubbard B.P., Sinclair D.A. (2014). Small molecule SIRT1 activators for the treatment of aging and age-related diseases. Trends Pharmacol. Sci..

[26] Brown V.A., Patel K.R., Viskaduraki M., Crowell J.A., Perloff M., Booth T.D., Vasilinin G., Sen A., Schinas A.M., Piccirilli G. (2010). Repeat dose study of the cancer chemopreventive agent resveratrol in healthy volunteers: safety, pharmacokinetics, and effect on the insulin-like growth factor axis. Cancer Res..

[27] Seyyedebrahimi S., Khodabandehloo H., Nasli Esfahani E., Meshkani R. (2018). The effects of resveratrol on markers of oxidative stress in patients with type 2 diabetes: a randomized, double-blind, placebo-controlled clinical trial. Acta Diabetol..

[28] Sergides C., Chirilă M., Silvestro L., Pitta D., Pittas A. (2016). Bioavailability and safety study of resveratrol 500 mg tablets in healthy male and female volunteers. Exp. Ther. Med..

[29] la Porte C., Voduc N., Zhang G., Seguin I., Tardiff D., Singhal N., Cameron D.W. (2010). Steady-State pharmacokinetics and tolerability of trans-resveratrol 2000 mg twice daily with food, quercetin and alcohol (ethanol) in healthy human subjects. Clin. Pharmacokinet..

[30] Johnson W.D., Morrissey R.L., Usborne A.L., Kapetanovic I., Crowell J.A., Muzzio M., McCormick D.L. (2011). Subchronic oral toxicity and cardiovascular safety pharmacology studies of resveratrol, a naturally occurring polyphenol with cancer preventive activity. Food Chem. Toxicol..

[31] Asensi M., Medina I., Ortega A., Carretero J., Bano M.C., Obrador E., Estrela J.M. (2002). Inhibition of cancer growth by resveratrol is related to its low bioavailability. Free Radic. Biol. Med..

[32] Smoliga J.M., Blanchard O. (2014). Enhancing the delivery of resveratrol in humans: if low bioavailability is the problem, what is the solution?. Molecules.

[33] Park E.J., Pezzuto J.M. (2015). The pharmacology of resveratrol in animals and humans. Biochim. Biophys. Acta.

[34] Gambini J., Ingles M., Olaso G., Lopez-Grueso R., Bonet-Costa V., Gimeno-Mallench L., Mas-Bargues C., Abdelaziz K.M., Gomez-Cabrera M.C., Vina J. (2015). Properties of Resveratrol: In Vitro and In Vivo Studies about Metabolism, Bioavailability, and Biological Effects in Animal Models and Humans. Oxid. Med. Cell. Longev..

[35] Johnson J.J., Nihal M., Siddiqui I.A., Scarlett C.O., Bailey H.H., Mukhtar H., Ahmad N. (2011). Enhancing the bioavailability of resveratrol by combining it with piperine. Mol. Nutr. Food Res..

[36] Pangeni R., Sahni J.K., Ali J., Sharma S., Baboota S. (2014). Resveratrol: review on therapeutic potential and recent advances in drug delivery. Expert Opin. Drug Deliv..

[37] Summerlin N., Soo E., Thakur S., Qu Z., Jambhrunkar S., Popat A. (2015). Resveratrol nanoformulations: challenges and opportunities. Int. J. Pharm..

[38] Peng R.M., Lin G.R., Ting Y., Hu J.Y. (2018). Oral delivery system enhanced the bioavailability of stilbenes: Resveratrol and pterostilbene. BioFactors.

[39] Walle T., Hsieh F., DeLegge M.H., Oatis J.E., Walle U.K. (2004). High absorption but very low bioavailability of oral resveratrol in humans. Drug Metab. Dispos..

[40] Andreadi C., Britton R.G., Patel K.R., Brown K. (2014). Resveratrol-sulfates provide an intracellular reservoir for generation of parent resveratrol, which induces autophagy in cancer cells. Autophagy.

[41] Calabrese E.J., Mattson M.P., Calabrese V. (2010). Dose response biology: the case of resveratrol. Hum. Exp. Toxicol..

[42] Juhasz B., Mukherjee S., Das D.K. (2010). Hormetic response of resveratrol against cardioprotection. Exp. Clin. Cardiol..

[43] Renaud S., de Lorgeril M. (1992). Wine, alcohol, platelets, and the French paradox for coronary heart disease. Lancet.

[44] Zou J.G., Wang Z.R., Huang Y.Z., Cao K.J., Wu J.M. (2003). Effect of red wine and wine polyphenol resveratrol on endothelial function in hypercholesterolemic rabbits. Int. J. Mol. Med..

[45] Wang Z., Zou J., Cao K., Hsieh T.C., Huang Y., Wu J.M. (2005). Dealcoholized red wine containing known amounts of resveratrol suppresses atherosclerosis in hypercholesterolemic rabbits without affecting plasma lipid levels. Int. J. Mol. Med..

[46] Lippi G., Franchini M., Favaloro E.J., Targher G. (2010). Moderate red wine consumption and cardiovascular disease risk: beyond the “French paradox”. Semin. Thromb. Hemost..

[47] Pastor R.F., Restani P., Di Lorenzo C., Orgiu F., Teissedre P.L., Stockley C., Ruf J.C., Quini C.I., Garcia Tejedor N., Gargantini R. (2017). Resveratrol, human health and winemaking perspectives. Crit. Rev. Food Sci. Nutr..

[48] Liberale L., Bonaventura A., Montecucco F., Dallegri F., Carbone F. (2017). Impact of Red Wine Consumption on Cardiovascular Health. Curr. Med. Chem..

[49] Frankel E.N., Waterhouse A.L., Kinsella J.E. (1993). Inhibition of human LDL oxidation by resveratrol. Lancet.

[50] Tadolini B., Juliano C., Piu L., Franconi F., Cabrini L. (2000). Resveratrol inhibition of lipid peroxidation. Free Radic. Res..

[51] Sebai H., Sani M., Ghanem-Boughanmi N., Aouani E. (2010). Prevention of lipopolysaccharide-induced mouse lethality by resveratrol. Food Chem. Toxicol..

[52] Avila P.R., Marques S.O., Luciano T.F., Vitto M.F., Engelmann J., Souza D.R., Pereira S.V., Pinho R.A., Lira F.S., De Souza C.T. (2013). Resveratrol and fish oil reduce catecholamine-induced mortality in obese rats: role of oxidative stress in the myocardium and aorta. Br. J. Nutr..

[53] Xia N., Daiber A., Forstermann U., Li H. (2017). Antioxidant effects of resveratrol in the cardiovascular system. Br. J. Pharmacol..

[54] Ye K., Ji C.B., Lu X.W., Ni Y.H., Gao C.L., Chen X.H., Zhao Y.P., Gu G.X., Guo X.R. (2010). Resveratrol attenuates radiation damage in Caenorhabditis elegans by preventing oxidative stress. J. Radiat. Res..

[55] Yin H., Si J., Xu H., Dong J., Zheng D., Lu X., Li X. (2014). Resveratrol-loaded nanoparticles reduce oxidative stress induced by radiation or amyloid-beta in transgenic Caenorhabditis elegans. J. Biomed. Nanotechnol..

[56] Jang M., Cai L., Udeani G.O., Slowing K.V., Thomas C.F., Beecher C.W., Fong H.H., Farnsworth N.R., Kinghorn A.D., Mehta R.G. (1997). Cancer chemopreventive activity of resveratrol, a natural product derived from grapes. Science.

[57] Das S., Das D.K. (2007). Anti-inflammatory responses of resveratrol. Inflamm. Allergy Drug Targets.

[58] Yeung F., Hoberg J.E., Ramsey C.S., Keller M.D., Jones D.R., Frye R.A., Mayo M.W. (2004). Modulation of NF-kappaB-dependent transcription and cell survival by the SIRT1 deacetylase. EMBO J..

[59] Kundu J.K., Shin Y.K., Surh Y.J. (2006). Resveratrol modulates phorbol ester-induced pro-inflammatory signal transduction pathways in mouse skin in vivo: NF-kappaB and AP-1 as prime targets. Biochem. Pharmacol..

[60] Kundu J.K., Shin Y.K., Kim S.H., Surh Y.J. (2006). Resveratrol inhibits phorbol ester-induced expression of COX-2 and activation of NF-kappaB in mouse skin by blocking IkappaB kinase activity. Carcinogenesis.

[61] Andrade J.M., Paraiso A.F., de Oliveira M.V., Martins A.M., Neto J.F., Guimaraes A.L., de Paula A.M., Qureshi M., Santos S.H. (2014). Resveratrol attenuates hepatic steatosis in high-fat fed mice by decreasing lipogenesis and inflammation. Nutrition.

[62] Leikert J.F., Rathel T.R., Wohlfart P., Cheynier V., Vollmar A.M., Dirsch V.M. (2002). Red wine polyphenols enhance endothelial nitric oxide synthase expression and subsequent nitric oxide release from endothelial cells. Circulation.

[63] Wallerath T., Deckert G., Ternes T., Anderson H., Li H., Witte K., Forstermann U. (2002). Resveratrol, a polyphenolic phytoalexin present in red wine, enhances expression and activity of endothelial nitric oxide synthase. Circulation.

[64] Klinge C.M., Blankenship K.A., Risinger K.E., Bhatnagar S., Noisin E.L., Sumanasekera W.K., Zhao L., Brey D.M., Keynton R.S. (2005). Resveratrol and estradiol rapidly activate MAPK signaling through estrogen receptors alpha and beta in endothelial cells. J. Biol. Chem..

[65] Xia N., Strand S., Schlufter F., Siuda D., Reifenberg G., Kleinert H., Forstermann U., Li H. (2013). Role of SIRT1 and FOXO factors in eNOS transcriptional activation by resveratrol. Nitric Oxide.

[66] Takahashi S., Nakashima Y. (2012). Repeated and long-term treatment with physiological concentrations of resveratrol promotes NO production in vascular endothelial cells. Br. J. Nutr..

[67] Gracia-Sancho J., Villarreal G., Zhang Y., Garcia-Cardena G. (2010). Activation of SIRT1 by resveratrol induces KLF2 expression conferring an endothelial vasoprotective phenotype. Cardiovasc. Res..

[68] Das S., Alagappan V.K., Bagchi D., Sharma H.S., Maulik N., Das D.K. (2005). Coordinated induction of iNOS-VEGF-KDR-eNOS after resveratrol consumption: a potential mechanism for resveratrol preconditioning of the heart. Vasc. Pharmacol..

[69] Lagouge M., Argmann C., Gerhart-Hines Z., Meziane H., Lerin C., Daussin F., Messadeq N., Milne J., Lambert P., Elliott P. (2006). Resveratrol improves mitochondrial function and protects against metabolic disease by activating SIRT1 and PGC-1alpha. Cell.

[70] Wang B., Sun J., Ma Y., Wu G., Tian Y., Shi Y., Le G. (2014). Resveratrol preserves mitochondrial function, stimulates mitochondrial biogenesis, and attenuates oxidative stress in regulatory T cells of mice fed a high-fat diet. J. Food Sci..

[71] Baur J.A., Pearson K.J., Price N.L., Jamieson H.A., Lerin C., Kalra A., Prabhu V.V., Allard J.S., Lopez-Lluch G., Lewis K. (2006). Resveratrol improves health and survival of mice on a high-calorie diet. Nature.

[72] Hardie D.G., Ross F.A., Hawley S.A. (2012). AMPK: a nutrient and energy sensor that maintains energy homeostasis. Nat. Rev. Mol. Cell Biol..

[73] Penumathsa S.V., Thirunavukkarasu M., Zhan L., Maulik G., Menon V.P., Bagchi D., Maulik N. (2008). Resveratrol enhances GLUT-4 translocation to the caveolar lipid raft fractions through AMPK/Akt/eNOS signalling pathway in diabetic myocardium. J. Cell. Mol. Med..

[74] da Luz P.L., Tanaka L., Brum P.C., Dourado P.M., Favarato D., Krieger J.E., Laurindo F.R. (2012). Red wine and equivalent oral pharmacological doses of resveratrol delay vascular aging but do not extend life Span in rats. Atherosclerosis.

[75] Jimenez-Gomez Y., Mattison J.A., Pearson K.J., Martin-Montalvo A., Palacios H.H., Sossong A.M., Ward T.M., Younts C.M., Lewis K., Allard J.S. (2013). Resveratrol improves adipose insulin signaling and reduces the inflammatory response in adipose tissue of rhesus monkeys on high-fat, high-sugar diet. Cell Metab..

[76] Fiori J.L., Shin Y.K., Kim W., Krzysik-Walker S.M., Gonzalez-Mariscal I., Carlson O.D., Sanghvi M., Moaddel R., Farhang K., Gadkaree S.K. (2013). Resveratrol prevents beta-cell dedifferentiation in nonhuman primates given a high-fat/high-sugar diet. Diabetes.

[77] Mattison J.A., Wang M., Bernier M., Zhang J., Park S.S., Maudsley S., An S.S., Santhanam L., Martin B., Faulkner S. (2014). Resveratrol prevents high fat/sucrose diet-induced central arterial wall inflammation and stiffening in nonhuman primates. Cell Metab..

[78] Wicinski M., Malinowski B., Weclewicz M.M., Grzesk E., Grzesk G. (2017). Anti-atherogenic properties of resveratrol: 4-week resveratrol administration associated with serum concentrations of SIRT1, adiponectin, S100A8/A9 and VSMCs contractility in a rat model. Exp. Ther. Med..

[79] Ungvari Z., Labinskyy N., Mukhopadhyay P., Pinto J.T., Bagi Z., Ballabh P., Zhang C., Pacher P., Csiszar A. (2009). Resveratrol attenuates mitochondrial oxidative stress in coronary arterial endothelial cells. Am. J. Physiol. Heart Circ. Physiol..

[80] Csiszar A., Labinskyy N., Pinto J.T., Ballabh P., Zhang H., Losonczy G., Pearson K., de Cabo R., Pacher P., Zhang C. (2009). Resveratrol induces mitochondrial biogenesis in endothelial cells. Am. J. Physiol. Heart Circ. Physiol..

[81] Ungvari Z., Bagi Z., Feher A., Recchia F.A., Sonntag W.E., Pearson K., de Cabo R., Csiszar A. (2010). Resveratrol confers endothelial protection via activation of the antioxidant transcription factor Nrf2. Am. J. Physiol. Heart Circ. Physiol..

[82] Aguirre L., Fernandez-Quintela A., Arias N., Portillo M.P. (2014). Resveratrol: anti-obesity mechanisms of action. Molecules.

[83] Zhao Y., Chen B., Shen J., Wan L., Zhu Y., Yi T., Xiao Z. (2017). The Beneficial Effects of Quercetin, Curcumin, and Resveratrol in Obesity. Oxid. Med. Cell. Longev..

[84] Fernandez-Quintela A., Milton-Laskibar I., Gonzalez M., Portillo M.P. (2017). Antiobesity effects of resveratrol: which tissues are involved?. Ann. N. Y. Acad. Sci..

[85] Kumar A., Kaundal R.K., Iyer S., Sharma S.S. (2007). Effects of resveratrol on nerve functions, oxidative stress and DNA fragmentation in experimental diabetic neuropathy. Life Sci..

[86] Do G.M., Jung U.J., Park H.J., Kwon E.Y., Jeon S.M., McGregor R.A., Choi M.S. (2012). Resveratrol ameliorates diabetes-related metabolic changes via activation of AMP-activated protein kinase and its downstream targets in db/db mice. Mol. Nutr. Food Res..

[87] Diaz M., Degens H., Vanhees L., Austin C., Azzawi M. (2016). The effects of resveratrol on aging vessels. Exp. Gerontol..

[88] Chen M.L., Yi L., Jin X., Liang X.Y., Zhou Y., Zhang T., Xie Q., Zhou X., Chang H., Fu Y.J. (2013). Resveratrol attenuates vascular endothelial inflammation by inducing autophagy through the cAMP signaling pathway. Autophagy.

[89] Marzetti E., Csiszar A., Dutta D., Balagopal G., Calvani R., Leeuwenburgh C. (2013). Role of mitochondrial dysfunction and altered autophagy in cardiovascular aging and disease: from mechanisms to therapeutics. Am. J. Physiol. Heart Circ. Physiol..

[90] Wohlgemuth S.E., Calvani R., Marzetti E. (2014). The interplay between autophagy and mitochondrial dysfunction in oxidative stress-induced cardiac aging and pathology. J. Mol. Cell Cardiol..

[91] Ren J., Zhang Y. (2018). Targeting Autophagy in Aging and Aging-Related Cardiovascular Diseases. Trends Pharmacol. Sci..

[92] Hayflick L. (1965). The Limited in Vitro Lifetime of Human Diploid Cell Strains. Exp. Cell Res..

[93] Campisi J. (1996). Replicative senescence: an old lives’ tale?. Cell.

[94] Vasto S., Candore G., Balistreri C.R., Caruso M., Colonna-Romano G., Grimaldi M.P., Listi F., Nuzzo D., Lio D., Caruso C. (2007). Inflammatory networks in ageing, age-related diseases and longevity. Mech. Ageing Dev..

[95] Munoz-Espin D., Serrano M. (2014). Cellular senescence: from physiology to pathology. Nat. Rev. Mol. Cell Biol..

[96] Schilder Y.D., Heiss E.H., Schachner D., Ziegler J., Reznicek G., Sorescu D., Dirsch V.M. (2009). NADPH oxidases 1 and 4 mediate cellular senescence induced by resveratrol in human endothelial cells. Free Radic. Biol. Med..

[97] Kao C.L., Chen L.K., Chang Y.L., Yung M.C., Hsu C.C., Chen Y.C., Lo W.L., Chen S.J., Ku H.H., Hwang S.J. (2010). Resveratrol protects human endothelium from H(2)O(2)-induced oxidative stress and senescence via SirT1 activation. J. Atheroscler. Thromb..

[98] Tang Y., Xu J., Qu W., Peng X., Xin P., Yang X., Ying C., Sun X., Hao L. (2012). Resveratrol reduces vascular cell senescence through attenuation of oxidative stress by SIRT1/NADPH oxidase-dependent mechanisms. J. Nutr. Biochem..

[99] Cao Z., Li Y. (2004). Potent induction of cellular antioxidants and phase 2 enzymes by resveratrol in cardiomyocytes: protection against oxidative and electrophilic injury. Eur. J. Pharmacol..

[100] Park D.W., Baek K., Kim J.R., Lee J.J., Ryu S.H., Chin B.R., Baek S.H. (2009). Resveratrol inhibits foam cell formation via NADPH oxidase 1- mediated reactive oxygen species and monocyte chemotactic protein-1. Exp. Mol. Med..

[101] Wang D., Uhrin P., Mocan A., Waltenberger B., Breuss J.M., Tewari D., Mihaly-Bison J., Huminiecki L., Starzynski R.R., Tzvetkov N.T. (2018). Vascular smooth muscle cell proliferation as a therapeutic target. Part 1: molecular targets and pathways. Biotechnol. Adv..

[102] Uhrin P., Wang D., Mocan A., Waltenberger B., Breuss J.M., Tewari D., Mihaly-Bison J., Huminiecki L., Starzynski R.R., Tzvetkov N.T. (2018). Vascular smooth muscle cell proliferation as a therapeutic target. Part 2: Natural products inhibiting proliferation. Biotechnol. Adv..

[103] Mizutani K., Ikeda K., Yamori Y. (2000). Resveratrol inhibits AGEs-induced proliferation and collagen synthesis activity in vascular smooth muscle cells from stroke-prone spontaneously hypertensive rats. Biochem. Biophys. Res. Commun..

[104] Kurin E., Atanasov A.G., Donath O., Heiss E.H., Dirsch V.M., Nagy M. (2012). Synergy study of the inhibitory potential of red wine polyphenols on vascular smooth muscle cell proliferation. Planta Med..

[105] Liu Y., Liu G. (2004). Isorhapontigenin and resveratrol suppress oxLDL-induced proliferation and activation of ERK1/2 mitogen-activated protein kinases of bovine aortic smooth muscle cells. Biochem. Pharmacol..

[106] El-Mowafy A.M., Alkhalaf M., Nassar N.N. (2009). Resveratrol reverses ET-1-evoked mitogenic effects in human coronary arterial cells by activating the kinase-G to inhibit ERK-enzymes. Int. J. Cardiol..

[107] Chen B., Xue J., Meng X., Slutzky J.L., Calvert A.E., Chicoine L.G. (2014). Resveratrol prevents hypoxia-induced arginase II expression and proliferation of human pulmonary artery smooth muscle cells via Akt-dependent signaling. Am. J. Physiol. Lung Cell. Mol. Physiol..

[108] Kwapiszewska G., Hoffmann J., Kovacs G., Stacher E., Olschewski A., Olschewski H. (2016). [Pulmonary (Arterial) Hypertension]. Pneumologie.

[109] Ma S.C., Zhang H.P., Jiao Y., Wang Y.H., Zhang H., Yang X.L., Yang A.N., Jiang Y.D. (2018). Homocysteine-induced proliferation of vascular smooth muscle cells occurs via PTEN hypermethylation and is mitigated by Resveratrol. Mol. Med. Rep..

[110] Rogers R.G., Otis J.S. (2017). Resveratrol-Mediated Expression of KLF15 in the Ischemic Myocardium is Associated with an Improved Cardiac Phenotype. Cardiovasc. Drugs Ther..

[111] Nording H.M., Seizer P., Langer H.F. (2015). Platelets in inflammation and atherogenesis. Front. Immunol..

[112] Wang Z., Huang Y., Zou J., Cao K., Xu Y., Wu J.M. (2002). Effects of red wine and wine polyphenol resveratrol on platelet aggregation in vivo and in vitro. Int. J. Mol. Med..

[113] Pace-Asciak C.R., Hahn S., Diamandis E.P., Soleas G., Goldberg D.M. (1995). The red wine phenolics trans-resveratrol and quercetin block human platelet aggregation and eicosanoid synthesis: implications for protection against coronary heart disease. Clin. Chim. Acta.

[114] Goh S.S., Woodman O.L., Pepe S., Cao A.H., Qin C., Ritchie R.H. (2007). The red wine antioxidant resveratrol prevents cardiomyocyte injury following ischemia-reperfusion via multiple sites and mechanisms. Antioxid. Redox Signal..

[115] Penumathsa S.V., Koneru S., Samuel S.M., Maulik G., Bagchi D., Yet S.F., Menon V.P., Maulik N. (2008). Strategic targets to induce neovascularization by resveratrol in hypercholesterolemic rat myocardium: role of caveolin-1, endothelial nitric oxide synthase, hemeoxygenase-1, and vascular endothelial growth factor. Free Radic. Biol. Med..

[116] Mukhopadhyay P., Mukherjee S., Ahsan K., Bagchi A., Pacher P., Das D.K. (2010). Restoration of altered microRNA expression in the ischemic heart with resveratrol. PLoS ONE.

[117] Rimbaud S., Ruiz M., Piquereau J., Mateo P., Fortin D., Veksler V., Garnier A., Ventura-Clapier R. (2011). Resveratrol improves survival, hemodynamics and energetics in a rat model of hypertension leading to heart failure. PLoS ONE.

[118] Ozdemir O.M., Gozkeser E., Bir F., Yenisey C. (2014). The effects of resveratrol on hyperoxia-induced lung injury in neonatal rats. Pediatr. Neonatol..

[119] Xu W., Zhao Y., Zhang B., Xu B., Yang Y., Wang Y., Liu C. (2015). Resveratrol attenuates hyperoxia-induced oxidative stress, inflammation and fibrosis and suppresses Wnt/beta-catenin signalling in lungs of neonatal rats. Clin. Exp. Pharmacol. Physiol..

[120] Torun A.C., Tutuncu S., Ustun B., Akdemir H.U. (2017). A Study of the Therapeutic Effects of Resveratrol on Blunt Chest Trauma-Induced Acute Lung Injury in Rats and the Potential Role of Endocan as a Biomarker of Inflammation. Inflammation.

[121] Virgili M., Contestabile A. (2000). Partial neuroprotection of in vivo excitotoxic brain damage by chronic administration of the red wine antioxidant agent, trans-resveratrol in rats. Neurosci. Lett..

[122] Wan D., Zhou Y., Wang K., Hou Y., Hou R., Ye X. (2016). Resveratrol provides neuroprotection by inhibiting phosphodiesterases and regulating the cAMP/AMPK/SIRT1 pathway after stroke in rats. Brain Res. Bull..

[123] Guo D., Xie J., Zhao J., Huang T., Guo X., Song J. (2018). Resveratrol protects early brain injury after subarachnoid hemorrhage by activating autophagy and inhibiting apoptosis mediated by the Akt/mTOR pathway. Neuroreport.

[124] Zhao P., Sui B.D., Liu N., Lv Y.J., Zheng C.X., Lu Y.B., Huang W.T., Zhou C.H., Chen J., Pang D.L. (2017). Anti-aging pharmacology in cutaneous wound healing: effects of metformin, resveratrol, and rapamycin by local application. Aging Cell.

[125] ClinicalTrials.gov. https://clinicaltrials.gov/ct2/results?cond=&term=resveratrol&cntry=&state=&city=&dist.

[126] Gresele P., Pignatelli P., Guglielmini G., Carnevale R., Mezzasoma A.M., Ghiselli A., Momi S., Violi F. (2008). Resveratrol, at concentrations attainable with moderate wine consumption, stimulates human platelet nitric oxide production. J. Nutr..

[127] Kennedy D.O., Wightman E.L., Reay J.L., Lietz G., Okello E.J., Wilde A., Haskell C.F. (2010). Effects of resveratrol on cerebral blood flow variables and cognitive performance in humans: a double-blind, placebo-controlled, crossover investigation. Am. J. Clin. Nutr..

[128] Sawda C., Moussa C., Turner R.S. (2017). Resveratrol for Alzheimer’s disease. Ann. N. Y. Acad. Sci..

[129] Timmers S., Konings E., Bilet L., Houtkooper R.H., van de Weijer T., Goossens G.H., Hoeks J., van der Krieken S., Ryu D., Kersten S. (2011). Calorie restriction-like effects of 30 days of resveratrol supplementation on energy metabolism and metabolic profile in obese humans. Cell Metab..

[130] Huang H., Chen G., Liao D., Zhu Y., Pu R., Xue X. (2016). The effects of resveratrol intervention on risk markers of cardiovascular health in overweight and obese subjects: a pooled analysis of randomized controlled trials. Obes. Rev..

[131] Mousavi S.M., Milajerdi A., Sheikhi A., Kord-Varkaneh H., Feinle-Bisset C., Larijani B., Esmaillzadeh A. (2018). Resveratrol supplementation significantly influences obesity measures: a systematic review and dose-response meta-analysis of randomized controlled trials. Obes. Rev..

[132] Tabrizi R., Tamtaji O.R., Lankarani K.B., Akbari M., Dadgostar E., Dabbaghmanesh M.H., Kolahdooz F., Shamshirian A., Momen-Heravi M., Asemi Z. (2018). The effects of resveratrol intake on weight loss: a systematic review and meta-analysis of randomized controlled trials. Crit. Rev. Food Sci. Nutr..

[133] Fogacci F., Tocci G., Presta V., Fratter A., Borghi C., Cicero A.F.G. (2018). Effect of resveratrol on blood pressure: A systematic review and meta-analysis of randomized, controlled, clinical trials. Crit. Rev. Food Sci. Nutr..

[134] Haghighatdoost F., Hariri M. (2018). Effect of resveratrol on lipid profile: An updated systematic review and meta-analysis on randomized clinical trials. Pharmacol. Res..

[135] Sahebkar A., Serban C., Ursoniu S., Wong N.D., Muntner P., Graham I.M., Mikhailidis D.P., Rizzo M., Rysz J., Sperling L.S. (2015). Lack of efficacy of resveratrol on C-reactive protein and selected cardiovascular risk factors--Results from a systematic review and meta-analysis of randomized controlled trials. Int. J. Cardiol..

[136] Kjaer T.N., Ornstrup M.J., Poulsen M.M., Stodkilde-Jorgensen H., Jessen N., Jorgensen J.O.L., Richelsen B., Pedersen S.B. (2017). No Beneficial Effects of Resveratrol on the Metabolic Syndrome: A Randomized Placebo-Controlled Clinical Trial. J. Clin. Endocrinol. Metab..

[137] Haghighatdoost F., Hariri M. (2018). Can resveratrol supplement change inflammatory mediators? A systematic review and meta-analysis on randomized clinical trials. Eur. J. Clin. Nutr..

[138] Koushki M., Dashatan N.A., Meshkani R. (2018). Effect of Resveratrol Supplementation on Inflammatory Markers: A Systematic Review and Meta-analysis of Randomized Controlled Trials. Clin. Ther..

[139] Tome-Carneiro J., Larrosa M., Yanez-Gascon M.J., Davalos A., Gil-Zamorano J., Gonzalvez M., Garcia-Almagro F.J., Ruiz Ros J.A., Tomas-Barberan F.A., Espin J.C. (2013). One-year supplementation with a grape extract containing resveratrol modulates inflammatory-related microRNAs and cytokines expression in peripheral blood mononuclear cells of type 2 diabetes and hypertensive patients with coronary artery disease. Pharmacol. Res..

[140] Knop F.K., Konings E., Timmers S., Schrauwen P., Holst J.J., Blaak E.E. (2013). Thirty days of resveratrol supplementation does not affect postprandial incretin hormone responses, but suppresses postprandial glucagon in obese subjects. Diabet. Med..

[141] Bhatt J.K., Thomas S., Nanjan M.J. (2012). Resveratrol supplementation improves glycemic control in type 2 diabetes mellitus. Nutr. Res..

[142] Movahed A., Nabipour I., Lieben Louis X., Thandapilly S.J., Yu L., Kalantarhormozi M., Rekabpour S.J., Netticadan T. (2013). Antihyperglycemic effects of short term resveratrol supplementation in type 2 diabetic patients. Evid. Based Complement. Altern. Med..

[143] Hausenblas H.A., Schoulda J.A., Smoliga J.M. (2015). Resveratrol treatment as an adjunct to pharmacological management in type 2 diabetes mellitus--systematic review and meta-analysis. Mol. Nutr. Food Res..

[144] Zhao H., Song A., Zhang Y., Shu L., Song G., Ma H. (2019). Effect of Resveratrol on Blood Lipid Levels in Patients with Type 2 Diabetes: A Systematic Review and Meta-Analysis. Obesity.

[145] Zhu X., Wu C., Qiu S., Yuan X., Li L. (2017). Effects of resveratrol on glucose control and insulin sensitivity in subjects with type 2 diabetes: systematic review and meta-analysis. Nutr. Metab..

[146] Liu Y., Ma W., Zhang P., He S., Huang D. (2015). Effect of resveratrol on blood pressure: a meta-analysis of randomized controlled trials. Clin. Nutr..

[147] Gliemann L., Schmidt J.F., Olesen J., Bienso R.S., Peronard S.L., Grandjean S.U., Mortensen S.P., Nyberg M., Bangsbo J., Pilegaard H. (2013). Resveratrol blunts the positive effects of exercise training on cardiovascular health in aged men. J. Physiol..

[148] Gliemann L., Olesen J., Bienso R.S., Schmidt J.F., Akerstrom T., Nyberg M., Lindqvist A., Bangsbo J., Hellsten Y. (2014). Resveratrol modulates the angiogenic response to exercise training in skeletal muscles of aged men. Am. J. Physiol. Heart Circ. Physiol..

[149] ClinicalTrials.gov Resveratrol and Exercise to Treat Functional Limnitation in Late Life. https://clinicaltrials.gov/ct2/results?cond=&term=resveratrol&cntry=&state=&city=&dist.

[150] Olesen J., Gliemann L., Bienso R., Schmidt J., Hellsten Y., Pilegaard H. (2014). Exercise training, but not resveratrol, improves metabolic and inflammatory status in skeletal muscle of aged men. J. Physiol..

[151] Smoliga J.M., Blanchard O.L. (2013). Recent data do not provide evidence that resveratrol causes ‘mainly negative’ or ‘adverse’ effects on exercise training in humans. J. Physiol..

[152] Smoliga J.M., Colombo E.S., Campen M.J. (2013). A healthier approach to clinical trials evaluating resveratrol for primary prevention of age-related diseases in healthy populations. Aging.

[153] Wright D.C. (2014). Exercise- and resveratrol-mediated alterations in adipose tissue metabolism. Appl. Physiol. Nutr. Metab..

[154] Bitterman J.L., Chung J.H. (2015). Metabolic effects of resveratrol: addressing the controversies. Cell. Mol. Life Sci..

[155] Belviranli M., Okudan N., Lamprecht M. (2015). Well-Known Antioxidants and Newcomers in Sport Nutrition: Coenzyme Q10, Quercetin, Resveratrol, Pterostilbene, Pycnogenol and Astaxanthin. Antioxidants in Sport Nutrition.

[156] Gliemann L., Nyberg M., Hellsten Y. (2016). Effects of exercise training and resveratrol on vascular health in aging. Free Radic. Biol. Med..

[157] Simioni C., Zauli G., Martelli A.M., Vitale M., Sacchetti G., Gonelli A., Neri L.M. (2018). Oxidative stress: role of physical exercise and antioxidant nutraceuticals in adulthood and aging. Oncotarget.

[158] Raj P., Louis X.L., Thandapilly S.J., Movahed A., Zieroth S., Netticadan T. (2014). Potential of resveratrol in the treatment of heart failure. Life Sci..

[159] Raj P., Zieroth S., Netticadan T. (2015). An overview of the efficacy of resveratrol in the management of ischemic heart disease. Ann. N. Y. Acad. Sci..

[160] Baczko I., Light P.E. (2015). Resveratrol and derivatives for the treatment of atrial fibrillation. Ann. N. Y. Acad. Sci..

[161] Barangi S., Hayes A.W., Karimi G. (2018). The more effective treatment of atrial fibrillation applying the natural compounds; as NADPH oxidase and ion channel inhibitors. Crit. Rev. Food Sci. Nutr..

[162] Beijers R., Gosker H.R., Schols A. (2018). Resveratrol for patients with chronic obstructive pulmonary disease: hype or hope?. Curr. Opin. Clin. Nutr. Metab. Care.

[163] Bradamante S., Barenghi L., Villa A. (2004). Cardiovascular protective effects of resveratrol. Cardiovasc. Drug Rev..

[164] Vang O., Ahmad N., Baile C.A., Baur J.A., Brown K., Csiszar A., Das D.K., Delmas D., Gottfried C., Lin H.Y. (2011). What is new for an old molecule? Systematic review and recommendations on the use of resveratrol. PLoS ONE.

[165] Tang P.C., Ng Y.F., Ho S., Gyda M., Chan S.W. (2014). Resveratrol and cardiovascular health-promising therapeutic or hopeless illusion?. Pharmacol. Res..

[166] Novelle M.G., Wahl D., Dieguez C., Bernier M., de Cabo R. (2015). Resveratrol supplementation: Where are we now and where should we go?. Ageing Res. Rev..

[167] Riccioni G., Gammone M.A., Tettamanti G., Bergante S., Pluchinotta F.R., D’Orazio N. (2015). Resveratrol and anti-atherogenic effects. Int. J. Food Sci. Nutr..

[168] Zordoky B.N., Robertson I.M., Dyck J.R. (2015). Preclinical and clinical evidence for the role of resveratrol in the treatment of cardiovascular diseases. Biochim. Biophys. Acta.

[169] Bonnefont-Rousselot D. (2016). Resveratrol and Cardiovascular Diseases. Nutrients.

[170] Oyenihi O.R., Oyenihi A.B., Adeyanju A.A., Oguntibeju O.O. (2016). Antidiabetic Effects of Resveratrol: The Way Forward in Its Clinical Utility. J. Diabetes Res..

[171] Cho S., Namkoong K., Shin M., Park J., Yang E., Ihm J., Thu V.T., Kim H.K., Han J. (2017). Cardiovascular Protective Effects and Clinical Applications of Resveratrol. J. Med. Food.

[172] Pezzuto J.M. (2018). Resveratrol: Twenty Years of Growth, Development and Controversy. Biomol. Ther..

[173] Ramirez-Garza S.L., Laveriano-Santos E.P., Marhuenda-Munoz M., Storniolo C.E., Tresserra-Rimbau A., Vallverdu-Queralt A., Lamuela-Raventos R.M. (2018). Health Effects of Resveratrol: Results from Human Intervention Trials. Nutrients.

[174] Wong R.H.X., Howe P.R.C. (2018). Resveratrol Counteracts Insulin Resistance-Potential Role of the Circulation. Nutrients.

